# Supplementary material for: Cold Atmospheric Plasma Jet Irradiation Decreases the Survival and the Expression of Oncogenic miRNAs of Oral Carcinoma Cells
Source: Int J Mol Sci. 2023 Nov 23;24(23):16662. doi: 10.3390/ijms242316662 (PMC10705903; doi:10.3390/ijms242316662)
Supplement: Supplementary file 1 [file ijms-24-16662-s001.zip › ijms-2682901-supplementary.pdf]

Supplementary Tables

Table S1. Materials used for fluorescence imaging

Reagent	Detection	Dilution	Incubation (°C /m)	Exciting wavelength (nm)	Emission wavelength (nm)	Cat no.	Manufacturer
**DCFDA**	ROS	1: 1000 1X buffer	37/45	458	535	ab113851	abcam, Cambridge, UK
**Annexin V-Alexa Fluor 488**	phosphati-dylserine	1: 20 in 1X Annexin V-binding buffer	25/15	490	525	V13241	Invitrogen
**DAPGreen**	autophago-some	1: 1000 in medium	37/30	488	530	D676	DOJINDO Lab, Kumamoto, Japan
**FerroOrange**	ferrous ion	1: 1000 in HBSS	37/30	543	580	F374	DOJINDO Lab
**Hoechst 33258**	DNA (stain of nucleus)	1: 1000 in 1X PBS	25/3	352	461	B1155	Sigma-Aldrich

Table S2. miRNA inhibitors or mimics

	Assay ID	Cat. no.	Manufacturer
Inhibitor control (Scr-2)	CM00200	AM17010	Ambion, Austin, TX, USA
hsa-miR-31 inhibitor	AM11465	AM17000	Ambion
Mimic control (Scr-1)		AM17110	Ambion
hsa-miR-21 mimic	MC10206	4464066	Ambion
hsa-miR-31 mimic	MC11465	4464066	Ambion
hsa-miR-146a mimic	MC10722	4464066	Ambion
hsa-miR-211 mimic	MC10168	4464066	Ambion

Table S3. TaqMan probes

	Cat. no.	Manufacturer
RNU6B	001903	Thermo Fisher Scientific
hsa-miR-21	000397	Thermo Fisher Scientific
hsa-miR-31	002279	Thermo Fisher Scientific
hsa-miR-125b	000449	Thermo Fisher Scientific
hsa-miR-134	000459	Thermo Fisher Scientific
hsa-miR-146a	000468	Thermo Fisher Scientific
hsa-miR-187	001193	Thermo Fisher Scientific
hsa-miR-211	000514	Thermo Fisher Scientific

Table S4 Antibodies

Antibody	Reactivity	Dilution	Cat. no.	Manufacturer
t-AKT (B-1)	Mouse	1:1000	sc-5298	Santa Cruz Biotech, Santa Cruz, CA, USA
pAKT (4051)	Mouse	1:1000	4051	Cell Signaling Tech, Danvers, MA, USA
t-ERK (K-23)	Rabbit	1:1000	sc-94	Santa Cruz Biotech
pERK	Rabbit	1:1000	9101	Cell Signaling Tech
GAPDH	Mouse	1:10000	sc-32233	Santa Cruz Biotech

Supplementary figures

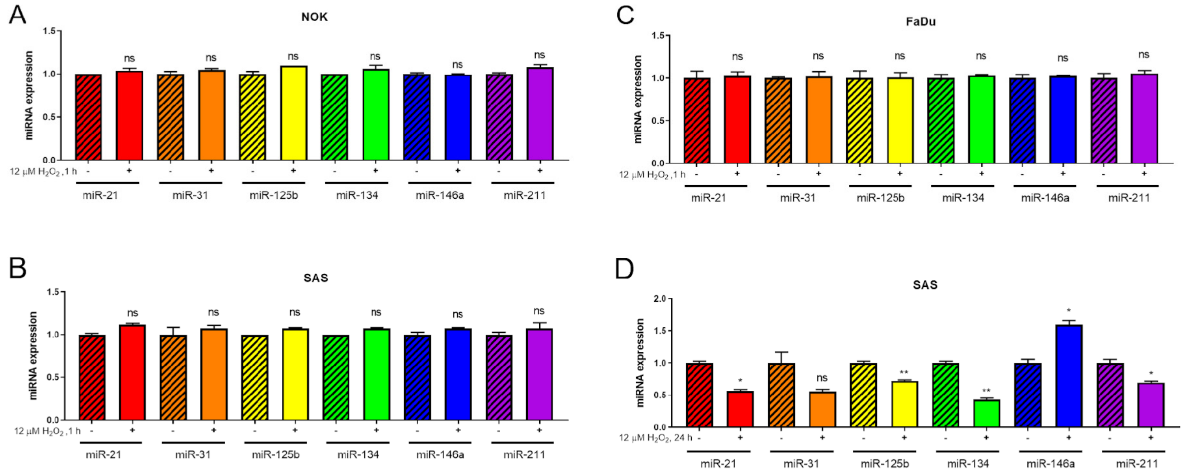

Figure S1. H_2O_2 treatment and miRNA expression. (A) NOK, (B, D) SAS, (C) FaDu. Cells are treated with 12.5 μM H_2O_2 for 1 h. (A - C) Culture medium is changed, and the miRNA expression is assayed 24 h later. (D) The miRNA expression in the cell is assayed 24 h later without changing the culture medium. Data shown are mean ± SE of duplicate or triplicate analysis. Mann-Whitney test. ns, not significant, *$p < 0.05$; **$p < 0.01$. Data in each panel derive from an individual experiment.

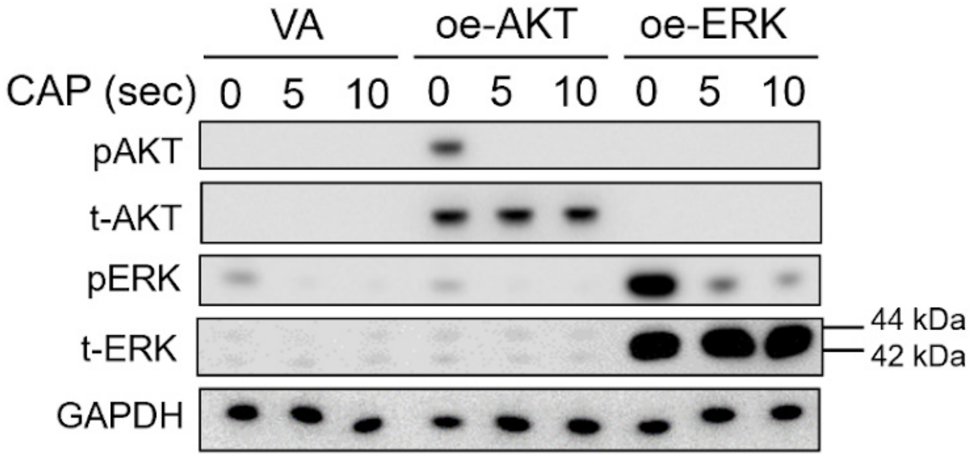

Figure S2. CAP irradiation inactivates AKT and ERK signals in SAS cells. SAS cells are transfected with plasmid in combination with CAP irradiation. The endogenous and exogenous expression along with phosphorylation of AKT and ERK 2 h after CAP treatment are analyzed. 10 s CAP irradiation elicits more profound ERK inactivation in relation to 5 s irradiation. The data are the representatives of two individual assays.

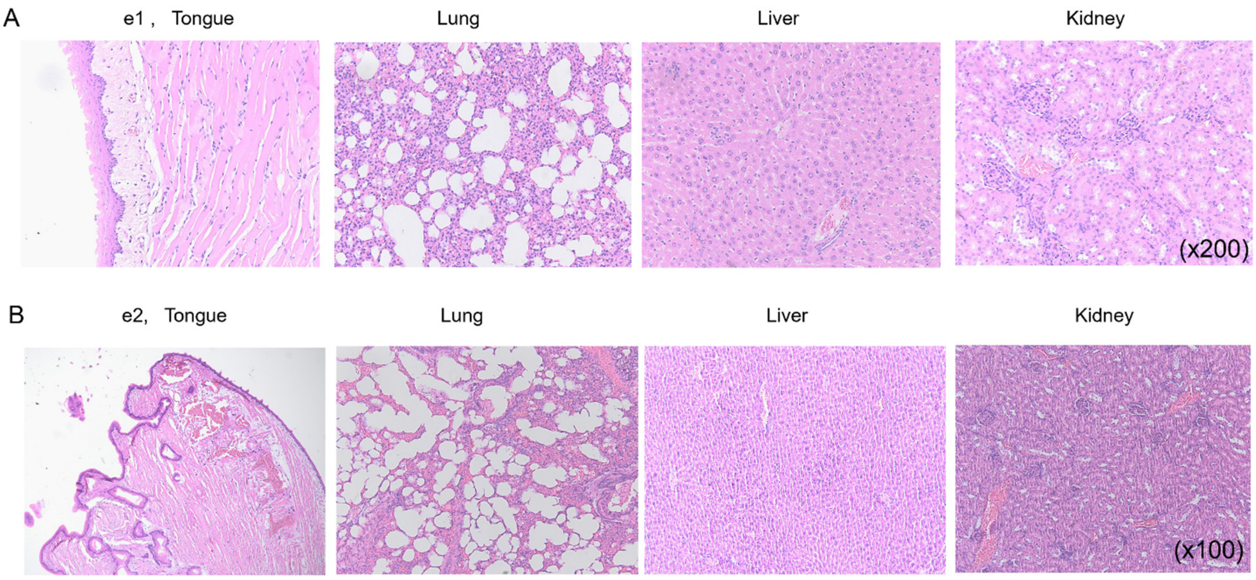

Figure S3. H&E-stained sections of tongue, lung, liver and kidney tissues harvested from mice receiving two-shot of 14 min CAP treatment. (A) e1 (B) e2. Number, magnification fold.

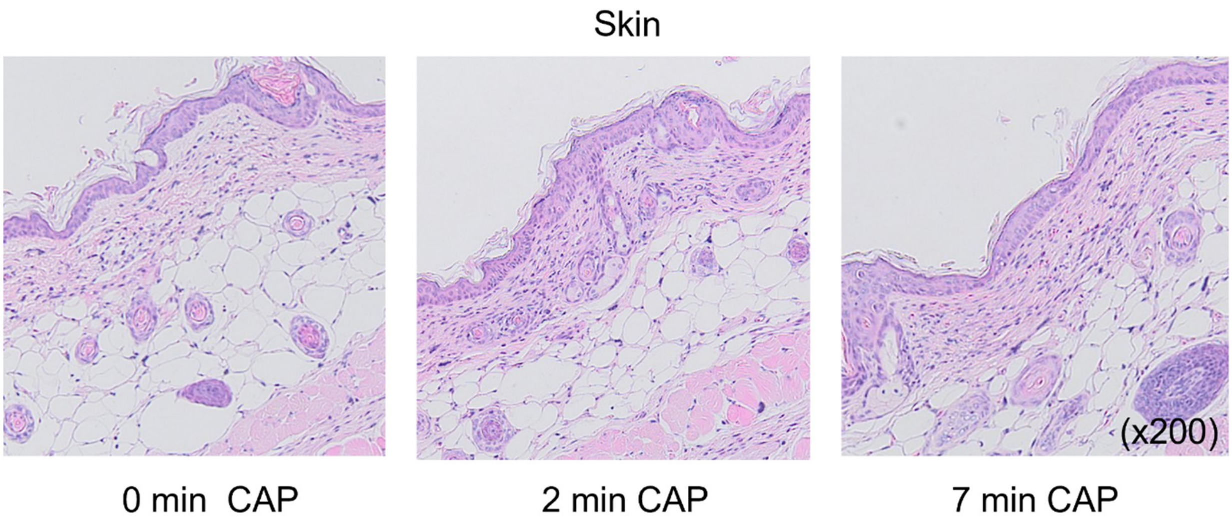

Figure S4. H&E-stained skin tissue sections from control, and those receiving 2 min or 7 min CAP treatment. No injury is noted in epidermis, dermis and hypodermis tissues. Number, magnification fold.

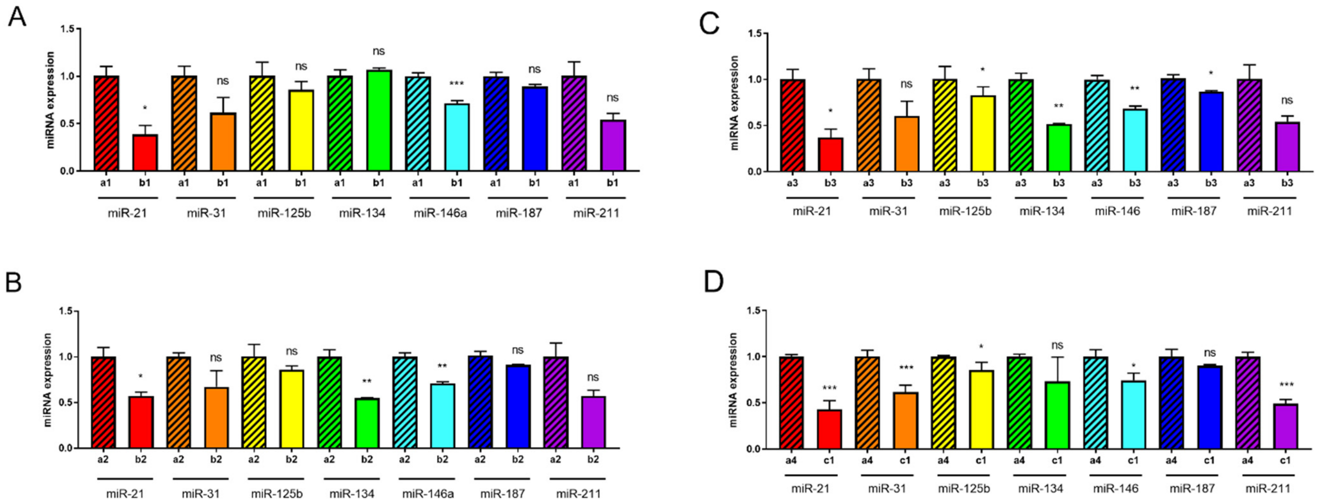

Figure S5. miRNA expression in four control tumors (a1- a4), three tumors receiving 10 min CAP treatment (b1 – b3 in A – C) and one tumor receiving 14 min CAP treatment (c1 in D). The data are organized in Figure 6F. Data shown are mean ± SE of duplicate or triplicate analysis. Mann-Whitney test. ns, not significant, **p* < 0.05; ***p*< 0.01. Data in each panel derive from an individual experiment.
